# Supplementary material for: Food consumption diversity and nutritional status among children aged 6–23 months in Indonesia: The analysis of the results of the 2018 Basic Health Research
Source: PLoS One. 2023 Mar 16;18(3):e0281426. doi: 10.1371/journal.pone.0281426 (PMC10019667; doi:10.1371/journal.pone.0281426)
Supplement: Attachment — Submitted filename: Response to Reviewers.docx [file pone.0281426.s002.docx]

Dear Dr. Resham B Khatri,

Thank you for your and the reviewers’ reply regarding our manuscript “Food consumption diversity and nutritional status among children aged 6–23 months in Indonesia: the analysis of the results of the 2018 Basic Health Research”. We are grateful for the reviewer’s comments and the positive valuation of our work. We have revised and modified the manuscript, as shown in the table below.

Reviewer #1

| Feedback | Response |
| --- | --- |
| “My only concern is that in the ethics statement, the author indicated as if he is the one who conduct the data collection which is not congruent with the statement in line 146 which shows that the author used secondary data from the basic health research. It is suggested that the reference of the ethics process is added, and permit to use the Riskesdas data is mentioned.” | Thank you for flagging this.  Yes, RISKESDAS survey was implemented by the Ministry of Health. We have edited the ethics statement accordingly. |

Reviewer #2

| Feedback | Responses |
| --- | --- |
| “I think thorough editing of the English language would enhance the manuscript and minimize grammatical and sentence errors.” | Thank you for your comments.  We have revised present tenses into past tenses. |
| “I noticed that authors used the present tense where it should have been be past; for instances; line no. 35, 66-67, 87, 100-please change these sentence to the past tense.” | Thank you for your input. We have edited these parts into past tenses. |
| “In line no. 64-65 reference is missing “ In the Southeast Asia region, the prevalence of stunting was the second highest 65 in Indonesia after Timor-Leste”[....Ref..]” | Thank you for pointing this out. We have added the reference number for this part. |
| “Please check the references again to make sure they adhere to journal standards. The manuscript requires some formatting work.” | Thank you for your suggestions. We have edited several parts of the reference list as PLOS ONE’s standards. |

All the changes above have improved the manuscript, and we hope it can meet PLOS ONE’s publication criteria.

Sincerely,

Authors: Omas Bulan Samosir, Dinda Srikandi Radjiman, and Flora Aninditya
